# Absence of Zika virus among pregnant women in Vietnam in 2008

**DOI:** 10.1186/s40794-023-00189-7

**Published:** 2023-03-01

**Authors:** Y.-C. Chui, D. Baud, A. Fahmi, B. Zumkehr, M. Vouga, L. Pomar, D. Musso, B. C. Thuong, M.P. Alves, M. Stojanovic

**Affiliations:** 1 Materno-Fetal and Obstetrics Research Unit, Department Woman-Mother-Child, Lausanne University Hospital, Lausanne, Switzerland; 2 Faculty of Biology and Medicine, University of Lausanne, Lausanne, Switzerland; 3 Institute of Virology and Immunology, Bern, Switzerland; 4 Department of Infectious Diseases and Pathobiology, Vetsuisse Faculty, University of Bern, Bern, Switzerland; 5 Graduate School for Cellular and Biomedical Sciences, University of Bern, Bern, Switzerland; 6 Aix Marseille Université, Institut de Recherche Pour Le Développement (IRD), Assistance Publique–Hôpitaux de Marseille, Service de Santé Des Armées, Vecteurs–Infections Tropicales et Méditerranéennes (VITROME), and Institut Hospitalo-Universitaire Méditerranée Infection, Marseille, France; 7 Laboratoire Eurofins Labazur Guyane, Eurofins, Cayenne, French Guiana; 8 Tu Du Hospital, District 1, Ho Chi Minh City, Vietnam; 9 Multidisciplinary Center for Infectious Diseases (MCID), University of Bern, Bern, Switzerland

**Keywords:** Zika virus, Placenta, Infection, Serology, Vietnam

## Abstract

**Background:**

Despite being first identified in 1947, Zika virus-related outbreaks were first described starting from 2007 culminating with the 2015 Latin American outbreak. Hypotheses indicate that the virus has been circulating in Asia for decades, but reports are scarce.

**Methods:**

We performed serological analysis and screened placental samples isolated in 2008 for the presence of Zika virus from pregnant women in Ho Chi Minh City (Vietnam).

**Results:**

None of the placental samples was positive for Zika virus. Four serum samples out of 176 (2.3%) specifically inhibited Zika virus, with variable degrees of cross-reactivity with other flaviviruses. While one of the four samples inhibited only Zika virus, cross-reactivity with other flaviviruses not included in the study could not be ruled out.

**Conclusion:**

Our results support the conclusion that the virus was not present among pregnant women in the Vietnamese largest city during the initial phases of the epidemic wave.

**Supplementary Information:**

The online version contains supplementary material available at 10.1186/s40794-023-00189-7.

## Introduction

Zika virus (ZIKV) is a mosquito-transmitted flavivirus that caused epidemics in Micronesia (2007), French Polynesia (2013) and, more recently in Latin America (2015) [1, 2]. Compared to other co-circulating arboviruses, including Dengue and Chikungunya viruses, ZIKV is associated with severe congenital anomalies in exposed foetuses such as microcephaly. ZIKV was first isolated in the Zika Forest, Uganda in 1947 [3]. Subsequently, the virus has diverged into two primary lineages, the prototypic African lineage and the Asian lineage. The latter was thought to be circulating on the Asian continent since the early 1950s [4], from which it migrated to the Yap State (Micronesia), causing the first major outbreak in 2017 [5]. ZIKV spread in the Pacific region, causing a second large outbreak in the French Polynesia [6, 7], before reaching South America between 2013 and 2015 [2], where the epidemic was declared a public health emergency of international concern by the WHO (from February to November 2016) [1]. ZIKV further spread to Central America, the Caribbean and reached the southern states of the USA [8, 9].

Sequence analyses confirmed that ZIKV isolates from the recent outbreaks were phylogenetically related and belonged to the Asian lineage [1]. Little is known regarding ZIKV circulation prior to the recent outbreaks. Serological evidence indicates that the virus was present on the Asian continent in the 1950s [10]. In Vietnam, ZIKV was first documented in 1954 [11] and this remained the only report until 2015 [12]. An outbreak was reported at the end of 2016, mainly in the southern part of the country, with 145 confirmed ZIKV cases [13]. Nevertheless, the effects of Zika presence in Vietnam remain largely unrecognised due to the lack of surveillance. Due to increased awareness from the recent outbreaks and associated severe adverse outcomes, several studies have proven its circulation in Asia [14–16].

In the present study, we intended to investigate the presence of ZIKV among pregnant women in Ho Chi Minh City (Vietnam) in 2008, the year following the first major outbreak in the Yap State (Micronesia). We utilized serum and placental samples obtained from pregnant women who delivered in 2008 [17]. The materno-fetal interface of the placenta plays a crucial role in the transmission of ZIKV to the fetus [18] and infection of placental tissue was previously reported in infected pregnant women, animal models of infection and in vitro assays [19–22].

## Methods

### Placental and serum samples

Placental and serum samples were collected at the Tu Du Hospital, Ho Chi Minh City (Vietnam) and stored at -80 °C upon reception [23]. Only samples for which the placental tissue was available were included in the present study, for a total of 176 samples. As for the original study, samples analysed here were collected from January to December 2008. All patients included in the study gave their written consent and utilization of the samples was approved by the local ethical committees of the Tu Du Hospital, Vietnam (clinical part) and the Lausanne University Hospital, Switzerland (experimental part).

### Flavivirus propagation and titration

Japanese encephalitis virus (JEV, CNS769_Laos_2009; GenBank accession number KC196115.1) was kindly provided by Remi Charrel, Aix-Marseille Université, France. West Nile virus (WNV, NY99–35, GenBank accession number DQ211652.1) was kindly provided by Martin Groschup, Friedrich-Loeffler-Institute, Germany. DENV-2 was kindly provided by Dr. Katja Fink, Singapore Immunology Network, Singapore). The low passage clinical isolate of Asian lineage ZIKV (PRVABC59, GenBank accession number KX377337) was obtained from Public Health England (PHE). Yellow fever virus (YFV, UVE/YFV/UNK/XX/Vaccinal strain 17D; GenBank accession number EU074025.1) was obtained at the European Virus Archive Global (EVAg). All flaviviruses were propagated in Vero cells (CCL-81, ATCC) cultured in DMEM (Gibco—Thermo Fisher Scientific, Reinach, Switzerland) supplemented with 10% FBS (Gibco) at 37 °C, 5% CO_2_. Flavivirus titers were determined in Vero cells using an immunoperoxidase assay using the anti-flavivirus group antigen antibody 4G2 (clone D1-4G2-4–15, ATCC, HB-112). Virus titers were calculated and expressed as 50% tissue culture infective dose per ml (TCID_50_/ml) using the Reed and Muench method [24].

### Serum neutralization assay

Serial twofold dilutions of heat-inactivated sera (30 min, 56 °C) were prepared in serum-free DMEM medium, with a starting dilution of 1:4 for ZIKV and 1:8 for JEV, WNV and YFV. Serial serum dilutions were incubated at 37 °C for 60 min with an equal volume of the corresponding virus to provide 100 PFU per 100 μl (1000 PFU per 100 μl in the case of DENV2). Serum-virus mixture was added to monolayers of Vero cells in 96-well flat-bottom tissue culture-treated microtiter plates (TPP) and incubated at 37 °C with 5% CO_2_ for two hours, after which DMEM containing 2% FBS 1% PenStrep (Gibco) was added to the wells. After 72 h, the culture plates were washed with 300 μl/well of cold PBS (Gibco) and fixed for 20 min at RT with 4% PFA. Then, the plates were washed once with 300 μl/well of 0.1% saponin (AppliChem GmbH, Darmstadt, Germany). A 100 μl/well of primary antibody mix was added and incubated for 30 min at 37 °C. The plates were washed twice with 200 μl/well of 0.1% saponin. Secondary antibody mix (rabbit anti-mouse HRP, Dako) was prepared in 0.3% saponin with a dilution factor of 1:250. A 100 μl/well of secondary antibody was added for 30 min at 37 °C in the dark. The plates were washed twice with 200 μl/well of 0.1% saponin and 80 μl/well of AEC substrate solution was added and incubated for 15 min at RT in the dark. When the plaques appeared with the desired coloration, the reaction was stopped with 100 μl/well of tap water. The microtiter plates were scanned with an ImmunoSpot analyzer (Cellular Technology Limited).

### Serological analysis

Serology for ZIKV was performed with the “Anti-Zika Virus ELISA (IgG)” kit (EI 2668–9601 G, EUROIMMUN Schweiz AG, Luzern, Switzerland), according to the manufacturer’s specifications. Confirmation of positive samples was performed using a custom designed flavivirus mosaic Indirect Immunofluorescence Test (IIFT) (EUROIMMUN Schweiz AG, Luzern, Switzerland), in which cells infected with Zika, Dengue (I-IV), West Nile, Yellow fever and Japanese encephalitis viruses were used to detect their antigens.

### Detection of ZIKV in placental samples

For the analysis of the presence of ZIKV in placental samples, approximately 25 mgs of tissue were homogenized using the Molecular Grinding Resin (G-Biosciences, St. Louis, MO, USA) and subsequently processed with NuceloSpin RNA II extraction kit for total RNA extraction according to the manufacturer’s protocol. Complementary DNA (cDNA) was obtained with the SuperScript II Reverse Transcriptase kit (Invitrogen Life Technologies, Carlsbad, CA, USA) by using Random Primer Hexamers (Invitrogen Life Technologies) and following the manufacturer’s instructions. ZIKV RNA levels were assessed by quantitative RT-PCR using the iTaq Universal Probes Supermix (Bio-Rad, Reinach, Switzerland) and the Rotor Gene 6000 thermocycler (Corbett Research, Sydney, Australia) as previously described [25]. As positive controls, placental homogenates were spiked with different inocula of ZIKV strain PRVABC59.

## Results

The 176 placental samples included in the study were tested for the presence of ZIKV RNA by RT-qPCR. None of the samples resulted positive, while viral RNA was successfully detected in all positive controls which were spiked with viral preparations.

Among the 176 samples included in the study, four (2.3%) were positive for ZIKV serology (ELISA analysis repeated twice for each sample). These samples were further tested for cross-reactivity against other flaviviruses, including Dengue (DENV, serotypes I-IV), West Nile (WNV), Yellow fever (YFV) and Japanese encephalitis (JEV) viruses. As depicted in Table 1, the four samples showed a high degree of cross-reactivity with all other flaviviruses. This can be further observed in Supplementary Fig. 1, with sample 2180 as an example.

**Table 1:** - Serological analysis of the four ZIKV-positive samples with other flaviviruses. Analysis cut-offs are shown (Pos)

	ZIKV	DENV-1	DENV-2	DENV-3	DENV-4	YFV	JEV	WNV
		IgG (Titer 1:)						
	Pos: ≥ 1:100						Pos: ≥ 1:10	
2028	≥ 3200	≥ 3200	≥ 3200	≥ 3200	≥ 3200	≥ 3200	≥ 3200	≥ 3200
2054	3200	10,000	10,000	10,000	10,000	1000	1000	1000
2087	10,000	32,000	32,000	32,000	32,000	10,000	10,000	10,000
2180	1000	10,000	10,000	10,000	3200	1000	1000	1000

To further characterise seropositive samples, we performed the serum neutralization assay (SNA), to assess serum ability to neutralize ZIKV virions. In parallel, SNA was done for DENV, JEV, WNV and YFV to monitor the extent of cross-reactivity sera samples (Table 2).

**Table 2:** – Serum neutralization assay performed with sera of seropositive samples

Sample	ZIKV	DENV	JEV	WNV	YFV
2028	1/64	1/1024	1/2048	neg	neg
2054	1/128	neg	neg	neg	neg
2087	neg	1/64	1/32	neg	neg
2180	neg	1/128	neg	neg	neg
Control 1^a^	neg	neg	neg	neg	1/256
Control 2^b^	neg	neg	1/64	neg	1/64

^a^Serum from vaccinated patient with YFV

^b^Serum from vaccinated patient with YFV, JEV

Serum sample 2054 was shown to specifically inhibit the infection by ZIKV, with no effect on the other flaviviruses included in the analysis (only one DENV serotype was tested). Cross-reactivity was observed for sample 2028, with the highest value for JEV. The remaining two serum samples (2087 and 2180) were not able to neutralize ZIKV.

## Discussion

Little is known about ZIKV circulation in Asia prior the recent major outbreak in Latin America. Samples used in this study were collected throughout the whole year, thus avoiding issues related with the seasonality of the presence of *Aedes* vectors in southern Vietnam. As expected, high levels of cross-reactivity between flaviviruses, which are endemic in Vietnam, were observed in our samples. In our sera collection from 176 pregnant women, samples from four patients (2.3%) were able to neutralize ZIKV in the serum neutralization assay. All the four pregnancies were uneventful. Interestingly, one sample did not show cross-reactivity with the flaviviruses tested here, although this could not exclude cross-reactivity with other flaviviruses, especially other DENV serotypes. While multiple reports indicate the circulation of ZIKV in Asia since the 1960s, very little is known about the extent and the characteristics of Asian clade strains prior to the recent outbreaks. Phylogenetic analyses showed that the ZIKV strain present in Micronesia in 2007 was related to a strain isolated in 2010 in Southeast Asia (Cambodia) [9]. It would be therefore of interest to perform analyses on samples isolated prior 2007 in Southeast Asian countries to gain more insight on the origin of the epidemic ZIKV clade. A 2017 retrospective study indicated that ZIKV was present in Vietnam prior to its identification in 2016 [26], however, the prevalence was very low (0.035%). Our results should be confirmed using a larger sample collection. Nevertheless, serological studies are complicated by the co-circulation of other flaviviruses in the endemic regions such as Vietnam and Southeast Asia more generally, as indicated by our results.

Importantly, ZIKV RNA could not be detected from placental samples included in this study, although transient infection of the placenta has been previously described in approximately 20% of infected women [17]. Nevertheless, we could not completely exclude the presence of the virus in the placental tissue, as only a fraction of the organ was used for RNA extraction. Moreover, we could not exclude degradation of viral RNA due to the relatively long period of storage.

Taken together, our results suggest that ZIKV was not present among pregnant women in Ho Chi Minh City (Vietnam) during the first epidemic episodes in the Pacific.

## Supplementary Information

**Additional file 1: Supplementary figure 1. **Cross-reactivity of Zika positive samples with other flaviviruses based on antigen detection in virus-infected cell lines. The results of sample 2180 are shown as an example, by using serum dilution at 1:1000. The mock control consists of mock infected cells.

## Data Availability

Data will be made available upon request to the authors.

## References

[1] Musso D, Ko AI, Baud D (2019). Zika virus infection - after the pandemic. N Engl J Med.

[2] Baud D, Gubler DJ, Schaub B, Lanteri MC, Musso D (2017). An update on Zika virus infection. The Lancet.

[3] Musso D, Gubler DJ (2016). Zika Virus. Clin Microbiol Rev.

[4] Duffy MR, Chen T-H, Hancock WT, Powers AM, Kool JL, Lanciotti RS (2009). Zika virus outbreak on Yap Island, Federated States of Micronesia. N Engl J Med.

[5] Musso D, Bossin H, Mallet HP, Besnard M, Broult J, Baudouin L (2017). Zika virus in French Polynesia 2013–14: anatomy of a completed outbreak. Lancet Infect Dis.

[6] Cauchemez S, Besnard M, Bompard P, Dub T, Guillemette-Artur P, Eyrolle-Guignot D (2016). Association between Zika virus and microcephaly in French Polynesia, 2013–15: a retrospective study. Lancet.

[7] Faria NR, Quick J, Claro IM, Thézé J, de Jesus JG, Giovanetti M (2017). Establishment and cryptic transmission of Zika virus in Brazil and the Americas. Nature.

[8] Metsky HC, Matranga CB, Wohl S, Schaffner SF, Freije CA, Winnicki SM (2017). Zika virus evolution and spread in the Americas. Nature.

[9] Haddow AD, Schuh AJ, Yasuda CY, Kasper MR, Heang V, Huy R (2012). Genetic characterization of Zika virus strains: geographic expansion of the asian lineage. PLoS Negl Trop Dis.

[10] Pond WL (1963). Arthropod-borne virus antibodies in sera from residents of South-East Asia. Trans R Soc Trop Med Hyg.

[11] Meltzer E, Lustig Y, Leshem E, Levy R, Gottesman G, Weissmann R (2016). Zika virus disease in traveler returning from Vietnam to Israel. Emerg Infect Dis.

[12] Chu D-T, Ngoc VTN, Tao Y (2017). Zika virus infection in Vietnam: current epidemic, strain origin, spreading risk, and perspective. Eur J Clin Microbiol Infect Dis.

[13] Umapathi T, Kam Y-W, Ohnmar O, Ng BCJ, Ng Y, Premikha M (2018). The 2016 Singapore Zika virus outbreak did not cause a surge in Guillain-Barré syndrome. J Peripher Nerv Syst.

[14] GeurtsvanKessel CH, Islam Z, Islam MB, Kamga S, Papri N, van de Vijver DAMC (2018). Zika virus and Guillain-Barré syndrome in Bangladesh. Ann Clin Transl Neurol.

[15] Ho HJ, Wong JGX, Mar Kyaw W, Lye DC, Leo YS, Chow A (2017). Diagnostic accuracy of parameters for Zika and dengue virus infections. Singapore Emerg Infect Dis.

[16] Ng DHL, Ho HJ, Chow A, Wong J, Kyaw WM, Tan A (2018). Correlation of clinical illness with viremia in Zika virus disease during an outbreak in Singapore. BMC Infect Dis.

[17] Pomar L, Lambert V, Madec Y, Vouga M, Pomar C, Matheus S (2019). Placental infection by Zika virus in French Guiana. Ultrasound in Obstet Gynecol: The Official Journal of the International Society of Ultrasound in Obstetrics and Gynecology.

[18] de Noronha L, Zanluca C, Burger M, Suzukawa AA, Azevedo M, Rebutini PZ (2018). Zika virus infection at different pregnancy stages: anatomopathological findings, target cells and viral persistence in placental tissues. Front Microbiol.

[19] Rabelo K, Souza LJ, Salomão NG, Oliveira ERA, Sentinelli L de P, Lacerda MS (2018). Placental inflammation and fetal injury in a rare Zika case associated with Guillain-Barré syndrome and abortion. Front Microbiol.

[20] Martines RB, Bhatnagar J, Keating MK, Silva-Flannery L, Muehlenbachs A, Gary J, Goldsmith C, et al. Notes from the Field: Evidence of Zika Virus Infection in Brain and Placental Tissues from Two Congenitally Infected Newborns and Two Fetal Losses--Brazil, 2015. MMWR Morb Mortal Wkly Rep. 2016;65(6):159-60. 10.15585/mmwr.mm6506e1. 10.15585/mmwr.mm6506e1 26890059

[21] Hirsch AJ, Roberts VHJ, Grigsby PL, Haese N, Schabel MC, Wang X, et al. Zika virus infection in pregnant rhesus macaques causes placental dysfunction and immunopathology. Nat Commun. 2018;9(1):263. 10.1038/s41467-017-02499-9. 10.1038/s41467-017-02499-9 PMC5772047 29343712

[22] Martinez Viedma M, Pickett B (2018). Characterizing the different effects of Zika virus infection in placenta and microglia cells. Viruses.

[23] Hornung S, Thuong BC, Gyger J, Kebbi-Beghdadi C, Vasilevsky S, Greub G (2015). Role of Chlamydia trachomatis and emerging Chlamydia-related bacteria in ectopic pregnancy in Vietnam. Epidemiol Infect.

[24] Richard V, Aubry M (2018). Method for simple and rapid concentration of Zika virus particles from infected cell-culture supernatants. J Virol Methods.

[25] Vielle NJ, Zumkehr B, García-Nicolás O, Blank F, Stojanov M, Musso D (2018). Silent infection of human dendritic cells by African and Asian strains of Zika virus. Sci Rep.

[26] Quyen NTH, Kien DTH, Rabaa M, Tuan NM, Vi TT, Van Tan L (2017). Chikungunya and Zika virus cases detected against a backdrop of endemic dengue transmission in Vietnam. Am J Trop Med Hyg.

