# Correction: Absence of Zika virus among pregnant women in Vietnam in 2008

**DOI:** 10.1186/s40794-023-00191-z

**Published:** 2023-04-06

**Authors:** Y.-C. Chiu, D. Baud, A. Fahmi, B. Zumkehr, M. Vouga, L. Pomar, D. Musso, B. C. Thuong, M. P. Alves, M. Stojanov

**Affiliations:** 1 Materno-Fetal and Obstetrics Research Unit, Department Woman-Mother-Child, Lausanne University Hospital, Lausanne, Switzerland; 2 Faculty of Biology and Medicine, University of Lausanne, Lausanne, Switzerland; 3 Institute of Virology and Immunology, Bern, Switzerland; 4 Department of Infectious Diseases and Pathobiology, Vetsuisse Faculty, University of Bern, Bern, Switzerland; 5 Graduate School for Cellular and Biomedical Sciences, University of Bern, Bern, Switzerland; 6 Aix Marseille Université, Institut de Recherche Pour Le Développement (IRD), Assistance Publique–Hôpitaux de Marseille, Service de Santé Des Armées, Vecteurs–Infections Tropicales et Méditerranéennes (VITROME), and Institut Hospitalo-Universitaire Méditerranée Infection, Marseille, France; 7 Laboratoire Eurofins Labazur Guyane, Eurofins, Cayenne, French Guiana; 8 Tu Du Hospital, District 1, Ho Chi Minh City, Vietnam; 9 Multidisciplinary Center for Infectious Diseases (MCID), University of Bern, Bern, Switzerland

**Correction: Trop Dis Travel Med Vaccines 9, 4 (2023)**

**https://doi.org/10.1186/s40794-023-00189-7**

After publication of this article [1], the authors reported that the author name Chiu was incorrectly written as Chui, and that the author name Stojanov was incorrectly written as Stojanovic.

The original article [1] has been corrected.
