# Supplementary material for: Absence of Zika virus among pregnant women in Vietnam in 2008
Source: Trop Dis Travel Med Vaccines. 2023 Mar 1;9:4. doi: 10.1186/s40794-023-00189-7 (PMC9976504; doi:10.1186/s40794-023-00189-7)
Supplement: Supplementary file 1 — Additional file 1: Supplementary figure 1. Cross-reactivity of Zika positive samples with other flaviviruses based on antigen detection in virus-infected cell lines. The results of sample 2180 are shown as an example, by using serum dilution at 1:1000. The mock control consists of mock infected cells. [file 40794_2023_189_MOESM1_ESM.docx]

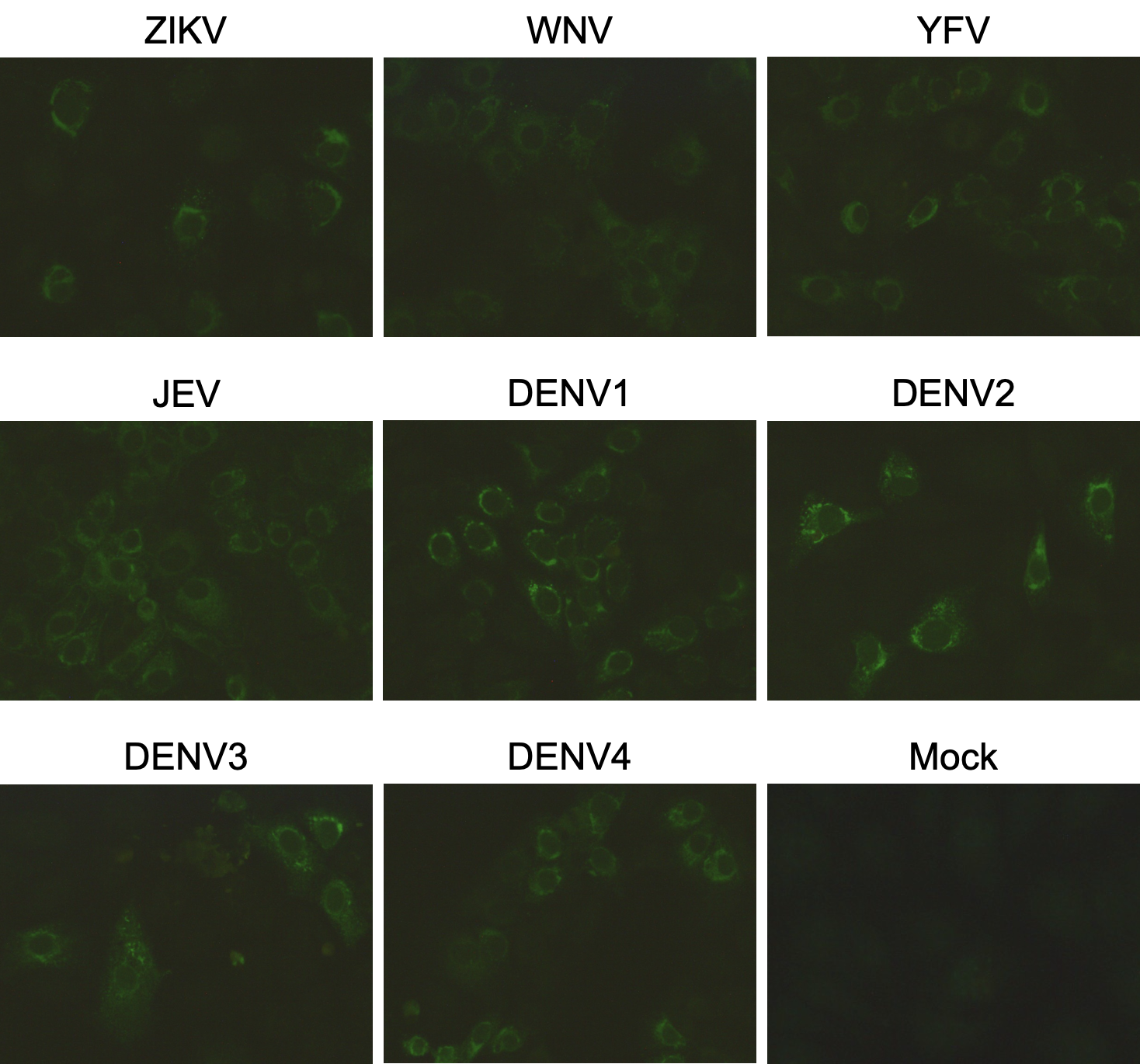


**Supplementary figure 1** – Cross-reactivity of Zika positive samples with other flaviviruses based on antigen detection in virus-infected cell lines. The results of sample 2180 are shown as an example, by using serum dilution at 1:1000. The mock control consists of mock infected cells.
